# Child exposure to animal feces and zoonotic pathogens in northwest Ecuador: A mixed-methods study

**DOI:** 10.1371/journal.pntd.0014019

**Published:** 2026-02-23

**Authors:** Viviana Albán, April M. Ballard, Kelsey J. Jesser, Gwenyth O. Lee, Joseph N.S. Eisenberg, Daniel Garzon-Chavez, Gabriel Trueba, Bethany A. Caruso, Karen Levy

**Affiliations:** 1 Instituto de Microbiología, Colegio de Ciencias Biológicas y Ambientales, Universidad San Francisco de Quito, Quito, Ecuador; 2 Department of Environmental and Occupational Health Sciences, University of Washington, Seattle, Washington, United States of America; 3 Gangarosa Department of Environmental Health, Emory University Rollins School of Public Health, Atlanta, Georgia, United States of America; 4 Department of Population Health Sciences, Georgia State University School of Public Health, Atlanta, Georgia, United States of America; 5 Rutgers Global Health Institute and Department of Biostatistics and Epidemiology, Rutgers University, New Brunswick, New Jersey, United States of America; 6 Department of Epidemiology, University of Michigan School of Public Health, Ann Arbor, Michigan, United States of America; 7 Colegio de Ciencias de la Salud, Universidad San Francisco de Quito, Quito, Ecuador; 8 Hubert Department of Global Health, Emory University Rollins School of Public Health, Atlanta, Georgia, United States of America; University of Virginia School of Medicine, UNITED STATES OF AMERICA

## Abstract

In low- and middle-income countries (LMICs), close cohabitation with animals and limited access to water, sanitation, and hygiene (WASH) infrastructure increase the risk of zoonotic enteric pathogen transmission to young children. This mixed-methods study combined (A) microbiological analysis of 120 animal fecal samples, and (B) go-along, semi-structured interviews with 35 mothers of children under two years across urban (n = 10), intermediate (n = 15), and rural (n = 10) communities in Ecuador to investigate: (Q1) What zoonotic enteric pathogens are present in animal feces and at what concentrations? (Q2) How are children exposed to animals and their feces? and (Q3) Which animals may serve as key sources of child exposure? Microbiological analysis revealed high prevalence and concentrations of zoonotic pathogens, most commonly *E. coli* aEPEC (57%), *Salmonella* sp. (36%), and *E. coli* STEC (25%), with frequent co-infections (33%) and concentrations (4.97-9.29 log10 gc/g) often exceeding infectious dose thresholds. Qualitative findings showed risks from free-roaming animals, poor feces management, and frequent child–animal contact, often indirectly through caregivers and siblings. Triangulation identified chickens and dogs as the most likely potential exposure sources due to their behaviors, proximity to children, and pathogen carriage. These findings highlight the need for targeted interventions to limit animal roaming, improve animal feces management, and increase caregiver awareness, while underscoring how mixed-methods approaches can help identify context-specific exposure pathways that should be considered when designing interventions.

## Author summary

In low-resource settings, such as northwestern coastal Ecuador, young children live in close contact with animals and often have limited access to clean water, toilets and safe play spaces. This raises concerns that microbial pathogens from animal feces may be an important, but often overlooked, source of illness. To understand how children may be exposed to these pathogens, we collected feces from common domestic animals around households. Using molecular methods, we found that more than 80% of samples contained microbes that can cause diarrhea in children, often at high levels and sometimes in combination. We also conducted qualitative interviews and walked through households with mothers, observing where animals spent time, where children played, and how animal feces were handled. By combining these two types of information, we identified chickens and dogs as key potential sources of exposure because they roam freely, defecate near living and play areas, carry multiple microbial pathogens, and often interact closely with children and mothers. Our findings suggest practical ways to reduce risk, such as limiting animal roaming near child play spaces and improving everyday feces cleanup in and around the home.

## Introduction

Zoonotic enteric pathogens are a major contributor to the global burden of diarrheal disease, particularly among children in low- and middle-income countries (LMICs) [1,2]. Young children are especially vulnerable to infections from zoonotic enteric pathogens due to their developing immune systems and frequent hand-to-mouth behaviors. Such infections during early life contribute not only to acute diarrhea but also to long-term consequences such as environmental enteric dysfunction, growth faltering, and cognitive deficits [3–5]. In LMICs, close cohabitation with animals, high densities of livestock, and inadequate access to water, sanitation, and hygiene (WASH) infrastructure, can create environments where animal fecal contamination is widespread [6,7]. Studies from multiple regions, including sub-Saharan Africa, South Asia, and Latin America, consistently show associations between the presence of animal feces in domestic environments and child diarrhea, stunting, and other adverse outcomes, underscoring the global relevance of this problem [8–11].

Animal fecal contamination in LMICs may be more extensive than human fecal contamination [12]. Approximately 29.7 x 10^9^ kg of animal feces are produced globally every year, almost four times the amount of human feces [12]. Although the specific fraction of infections attributable to animal sources remains poorly quantified, animal feces is a major reservoir for many pathogens capable of infecting humans, including *Campylobacter* spp., non-typhoidal *Salmonella*, enteropathogenic *E. coli,* and *Cryptosporidium* spp. These four pathogens alone account for 28.3% of the global diarrheal deaths among children under age five [1]. Major transmission pathways include contamination of water through runoff, contamination of soils and crops through animal defecation or fertilizer use, and exposure through animal-derived foods. Additional pathways include unsafe feces disposal, contamination of household surfaces, and direct contact with fecal matter [2]. Characterizing human exposure to animal feces therefore requires understanding both the environmental source and distribution of pathogens and the child behaviors that mediate exposure [2,13].

Despite growing evidence of the burden and transmission of zoonotic enteric pathogens, interventions to reduce child exposure remain insufficiently targeted. Vaccines and WASH interventions are common strategies for reducing enteric disease burdens. However, vaccines are not available for all enteric pathogens [14], and for those that exist, uptake can be low in LMICs [15]. WASH interventions can reduce the risk of child diarrhea [16], but large scale randomized trials have not resulted in the anticipated improvements in child health outcomes [9,17,18]. One possible explanation is that most WASH interventions did not adequately address animal fecal contamination [14,15], and as a result, research at the intersection of WASH and animals has grown. However, a critical gap persists: few studies have jointly investigated both (a) the specific animal sources of zoonotic enteric pathogens and (b) the child behaviors that contribute to exposure pathways. Without integrating microbiological and behavioral data, interventions may miss the highest-impact points of prevention.

This mixed-methods study fills this gap by analyzing zoonotic enteric pathogen data alongside child and caregiver behaviors to characterize zoonotic enteric pathogen exposure pathways among children under two years of age along an urban-rural gradient in northwestern Ecuador, an area known for high enteric pathogen transmission. The two-week prevalence of enteropathogenic *E. coli* infections and diarrhea among children under age five in Ecuador has been estimated to be around 25% and 9%, respectively [19]. Our work addressed three research questions: (Q1) What zoonotic enteric pathogens are present in animal feces and at what concentrations? (Q2) How are children exposed to animals and their feces? and (Q3) Which animals may serve as key sources of child exposure to zoonotic pathogens, based on observed behaviors and fecal contamination? We also explored how these risks differ across the urban–rural gradient, though the primary focus of the study is on overall patterns.

## Methods

### Study design and setting

**Fig 1:**
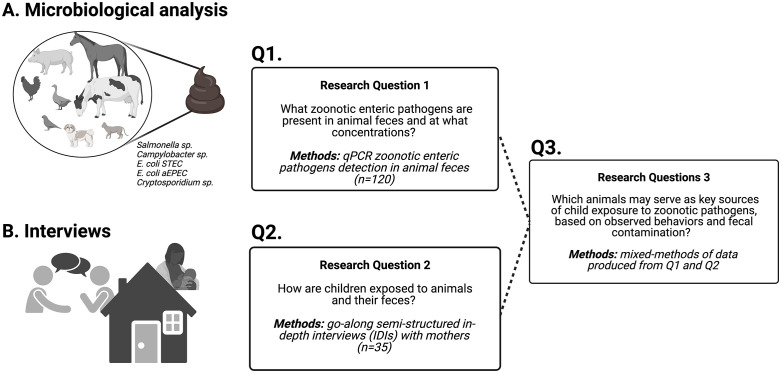
Integration of qualitative and microbiological methods (convergent mixed-methods design) to investigate zoonotic pathogen exposure in children. We used: **A.** Microbiological methods (qPCR) for the detection of zoonotic enteric pathogens to answer Q1 **B.** Qualitative methods, go-along semi-structured in-depth interviews (IDIs), to answer Q2. **C.** Triangulation of qualitative and microbial data to answer Q3. Created in BioRender. Alban, **V.** (2026) https://BioRender.com/7j6blpq [20].

We used a convergent mixed methods design consisting of three components: (A) microbiological analyses of animal feces collected in and around participant households, to identify what zoonotic enteric pathogens are present and at what concentrations (Q1); (B) go-along semi-structured in-depth interviews (IDIs), a hybrid between participant observation and interviewing [18,19], to understand how children are exposed to animals and their feces (Q2); and (C) integration of microbiological and qualitative data to identify the most likely potential animal sources of child exposure (Q3) (Fig 1). This approach combines microbiological evidence with contextual insights on child–animal and child–environment interactions to provide a holistic view of exposure pathways.

We conducted this study between June and August 2019 in Esmeraldas Province, northwestern Ecuador, across communities along an urban–rural gradient: (i) Esmeraldas city (‘Urban’ community; population ~150,000); (ii) Borbón (‘Intermediate’ community, a town that serves as a center for commercial activities; population ~4,500), (iii) Maldonado (‘Rural road’ community; population ~2,000), and (iv) Santo Domingo and Colón (‘Rural river’ communities; Santo Domingo: population ~500; Colón: population ~920). Santo Domingo and Colón are approximately 3.5 hours by boat from Borbón and are generally inaccessible by road, except for limited seasonal access. Due to small sample sizes, we combined the ‘Rural road’ and ‘Rural river’ communities into a single ‘Rural’ communities category (S1 Fig).

### Ethics

The Institutional Review Boards at Emory University (Atlanta, USA; STUDY00010353) and Universidad San Francisco de Quito (Quito, Ecuador; 2018-022M) approved the study protocol. Before data collection, we informed participants of the study aims and obtained written consent. Interviews were recorded with participant permission.

### Sample selection and size

To understand how children are exposed to animals and their feces, we recruited mothers of children under two who owned at least one animal. We focused on this age group because infants become increasingly mobile and vulnerable to environmental exposures [5,13,21], and early childhood is a critical developmental period when repeated enteric infections can have lasting impacts on health and development [3,4].

Research assistants identified households with children under two and animal ownership by walking through communities. Using purposive sampling, we included households with diverse types and numbers of animals to represent each community. We aimed to conduct 30 IDIs (10 per community), with sample size informed by evidence that data saturation is typically reached within 9–17 qualitative interviews [22].

### Data collection and management

#### Microbiological data.

To identify what zoonotic enteric pathogens are present in what types of animal feces and at what concentrations, we collected 120 animal fecal samples from in and around the households of the mothers who were interviewed. We targeted visibly fresh fecal samples from areas where animals typically rest or roam (e.g., patios, yards, kitchen, and corrals). Animal species were identified by direct observation and confirmed with mothers. Fecal samples for the following domestic animals were collected: chickens (n = 28), dogs (n = 21), cats (n = 6), pigs (n = 21), cows (n = 14), horses (n = 13), and other birds (ducks and parrots) (n = 14).

For each animal fecal sample, we used sterile gloves and spatulas to collect 5–10 g of feces into pre-labeled sterile containers. Samples were taken from the interior of the stool, avoiding the superficial layer and any portion in direct contact with the ground. Samples were stored on ice and transported to nearby field labs, where within six hours we aliquoted ~1 g into four 1 ml cryovials and flash-froze them in liquid nitrogen. Samples were then transported to the Universidad San Francisco de Quito (USFQ) and stored at –80 °C.

We extracted genomic DNA from animal fecal samples using the PowerSoil DNA Isolation Kit (MoBio Laboratories, Inc., Carlsbad, CA, US) following the manufacturer’s protocol. Quantitative PCR (qPCR) was used to detect and quantify atypical enteropathogenic *E. coli* (aEPEC), Shiga toxin-producing *E. coli* (STEC), *Salmonella* sp., and *Campylobacter* sp., while *Cryptosporidium* sp. was identified using an ELISA assay. We selected pathogens with a substantial burden of disease and potentially important transmission in animal feces (*Campylobacter* sp., non-typhoidal *Salmonella*, *Cryptosporidium* sp.), an unquantified burden of disease and potentially important transmission in animal feces (STEC), or with a substantial burden of disease and insufficient evidence of transmission in animal feces (aEPEC) [1]. Assay gene targets along with primers, probes, and standard gblock (synthetic gene fragments containing all enteric pathogens target genes) sequences are presented in S1 Table. We generated standard curves from serial 10-fold dilutions of a gBlock standard containing the target DNA sequences, with concentrations ranging from 10^6^ to 10^2^ gene copies per reaction (S2 Fig). We included a standard curve on each qPCR assay plate and prepared a fresh gblock dilution series daily.

We ran duplicate standards and samples on a real-time PCR system (CFX96, Bio-Rad, USA) in a final reaction volume of 20 uL. Each reaction included 10 ul 2X Taqman Universal PCR Master Mix (Applied biosystems, Life Technologies Corporation, Carlsband, CA, US), 1 uM of each forward and reverse primer, 0.1 uM of probe, and 4 ul of DNA template. Cycling conditions were as follows: 50°C for 2 min, 95°C for 10 min, and 40 cycles of 95°C for 15 sec, and 55°C for 1 min (for *invA* [23], *eae* [24], *stx1* [24] and *stx2* [24])/ 60°C for 1 min (for *bfpA* [23] and *cadF* [23]). We included non-template controls (NTCs) and negative extraction controls (NECs) in each run. We assessed qPCR inhibition by spiking each DNA extract with1 x 10^6^ copies of a gblock containing a 220-bp artificial inhibition control (IC) sequence [25]. We amplified the IC using a SYBR green qPCR assay and confirmed specificity by melt curve analysis. No inhibition of qPCR amplification was detected. We determined the presence or absence of *Cryptosporidium* sp. using the RIDASCREEN *Cryptosporidium* enzyme immunoassay (EIA), following the manufacturer’s instructions. We included positive and negative controls provided in the kit on each ELISA plate. Although RIDASCREEN *Cryptosporidium* sp. EIA was originally developed for human stool, its performance has been evaluated in animal fecal samples, showing a moderate agreement (kappa: 54%) with PCR methods [26].

#### Qualitative data.

After obtaining consent, three trained Ecuadorian interviewers (two women, including author VAM, and one man) and a note-taker (non-Ecuadorian author AMB) conducted go-along, semi-structured IDIs. The guide covered animal ownership, environmental characteristics, child behaviors and interactions with animals, animal feces, and feces-contaminated soil, with probes used as needed (S1 File). Mothers showed interviewers where animals lived and spent time. If observation was not possible, a traditional in-depth interview was conducted instead. Participants also completed a 10-question survey on maternal and child demographics, household water and sanitation, and animal ownership. With permission, interviews were audio recorded and lasted 15–60 minutes. Interviewers collected detailed notes, observations, and animal photographs (without human faces). After each interview, AMB and the team debriefed to review flow, discuss emerging themes, and document key observations to refine data collection in real time [27].

### Data analysis

#### Microbiological data.

Raw qPCR data were processed to determine presence/absence and abundance of targeted pathogens (Q1). Gene abundance was quantified relative to a mean standard curve, calculated from four curves run with each assay, and analyzed according to MIQE guidelines [28]. A completed MIQE checklist summarizing assay design, validation and run conditions for all qPCR assays is provided in S2 Table. We defined the assay limits of detection (LoD) as the lowest standard concentration that met two criteria: a standard deviation <1 for replicates and >95% replicate detection. We calculated assay limits of quantification (LoQ) using the LoD Cq value and its standard deviation (σ) as follows: CtLoQ = CtLoD – 2(σLoD). We summarized assay LoD, efficiency, linear dynamic range, and standard curve R^2^ values and slopes for each assay in S1 Table. NTCs and NECs included on each plate showed no amplification at 40 cycles.

We considered a sample Detectable and Quantifiable (DQ) if the target amplified in both duplicate qPCR reactions and the mean Cq value was both>LoQ and between the highest and lowest dilutions on the standard curve. We classified samples Detectable but Not Quantifiable (DNQ) or Not Detected (ND) otherwise [26]. DQ and DNQ results were considered positive (present), while ND results were considered negative (absent) for prevalence analyses. For the *Cryptosporidium* sp. ELISA assay, we established a cut-off value by adding 0.15 extinction units to the negative control measurement. A sample was considered positive for *Cryptosporidium* sp. if its extinction rate exceeded the cut-off by more than 10%.

We performed all statistical analyses and data visualization using R version 4.4.1. Pathogen prevalence was calculated as the number of positive samples divided by the total tested. Chi-square tests assessed whether pathogen frequencies differed across communities and animal species, while Wilcoxon rank-sum tests compared pathogen concentrations (non-normally distributed) between communities.

To examine whether specific pathogens tended to occur together in animal feces more or less often than expected by chance, we conducted a probabilistic species co-occurrence analysis [29]. For each pair of pathogens, we calculated the expected number of co-occurrences under an assumption of independence, given the marginal prevalence of each pathogen, and then compared the observed co-occurrence to this expectation using a probabilistic framework [29]. Pathogen pairs with observed co-occurrence significantly greater than expected were interpreted as showing positive co-occurrences, whereas pairs with significantly fewer co-occurrences than expected were interpreted as negative co-occurrence. This analysis complements our descriptive prevalence estimate by identifying non-random clustering of pathogens within samples, which may reflect shared hosts, transmission pathways, or environmental reservoirs.

#### Qualitative data.

To understand how children are exposed to animals and their feces (Q2), we conducted thematic analysis using Word and Excel to organize and review qualitative materials. Data sources included observational and interview notes, audio recordings, photographs, detailed interview summaries, and community-level contextual information. Following data collection, observational and interview notes, audio transcripts, and photographs were first reviewed together to construct a detailed summary of each interview. These summaries integrated descriptive accounts with reflexive notes made throughout fieldwork, which documented researchers’ observations, interpretations, and evolving questions. We then iteratively revisited the original notes, audio recordings, and photographs to expand, refine, and cross-check each summary.

Next, interview summaries, reflexive notes, and reviews of the original notes, recordings, and photographs were used to construct expanded community-level notes and preliminary analyses. From these materials, we developed comprehensive community profiles that synthesized information on animal ownership, animal husbandry, animal health, feces exposure practices, child behaviors, and maternal perceptions and norms. These profiles served as analytic scaffolds that allowed us to examine patterns both within and across communities.

We then repeated this process iteratively to move from community-level synthesis to overarching cross-community themes. Throughout the analysis, we wrote analytic memos to document emerging themes, relationships between concepts, and evolving interpretations. To enhance rigor and validity, authors AMB and VAM reviewed and discussed the thematic interpretation to ensure consistency and to confirm that conclusions were grounded in the full data set. Themes were revisited multiple times during analysis, following standard qualitative analytic procedures.

#### Triangulation of qualitative and microbiological data.

To identify which animals may serve as sources of child exposure to zoonotic pathogens (Q3), we triangulated findings from the microbiological analyses (Q1) and qualitative interviews (Q2). Specifically, we compared reported and observed patterns of child–animal interactions and environmental conditions with the prevalence of zoonotic pathogens detected in animal feces. This included linking behavioral themes, such as proximity of animals to living areas and child caregiving practices, to the detection of pathogens in different animal species across communities.

We integrated these streams of data to identify alignments and discrepancies between child behaviors and microbial contamination patterns, allowing us to draw connections between specific animals, exposure contexts, and settings. For example, animals frequently observed near children or in shared household spaces were examined for their pathogen carriage to assess potential child exposure. Authors AMB and VAM jointly produced the thematic findings and iteratively reviewed and discussed them to ensure interpretive rigor and consistency.

To explore if and how animal-related risks vary along the urban-rural gradient for the three research questions, we stratified our results by community and identified overlap and divergence. For microbiological data, this included chi-square tests, Wilcoxon rank-sum tests, along with prevalence plots, boxplots, and summary tables. For qualitative data, this meant comparing key themes by iteratively reviewing notes, transcripts of audio recordings, photographs, summaries, and profiles.

## Results

### Sample characteristics

The final qualitative sample included 35 mother–child dyads. Most households owned dogs (63%) and/or cats (60%) (Table 1). Animal ownership diversity increased with rurality: urban households owned 1–2 species, intermediate 1–4, and rural 1–6. Mothers were, on average, 26 years old (range: 18–43 years) and children were 13 months old (range: 2–23 months). Urban households used purchased (50%) and piped (50%) water for drinking and household toilets or latrines (100%) for sanitation, while those in the intermediate community largely used purchased water (73%) along with well or tubewell water (7%), rainwater (7%), and piped water (13%), but also largely used household toilets or latrines (93%). Rural households solely used rainwater (70%) or river water (30%) for drinking and used varied sanitation, including household toilets or latrines (50%), public or community latrines (20%), or a neighbor’s toilet or latrine (30%). Additional household and demographic details are provided in S3 Table.

**Table 1:** Household animal ownership by animal species and community.

Animal type	Rural (n = 10)	Intermediate (n = 15)	Urban (n = 10)	Overall (n = 35)
**Chickens**	3 (30%)	6 (40%)	4 (40%)	13 (37%)
**Dogs**	5 (50%)	11 (73%)	6 (60%)	22 (63%)
**Cats**	6 (60%)	9 (60%)	6 (60%)	21 (60%)
**Pigs**	1 (10%)	3 (20%)	0 (0%)	4 (11%)
**Horses/Donkeys/Cows**	2 (20%)	0 (0%)	0 (0%)	2 (6%)
**Other birds**	0 (0%)	2 (13%)	0 (0%)	2 (6%)

Percentages indicate the proportion of households reporting ownership of each animal species within each community. “Overall” reflects data combined across all study sites.

### (Q1) What zoonotic enteric pathogens are present in animal feces and at what concentrations?

**Fig 2:**
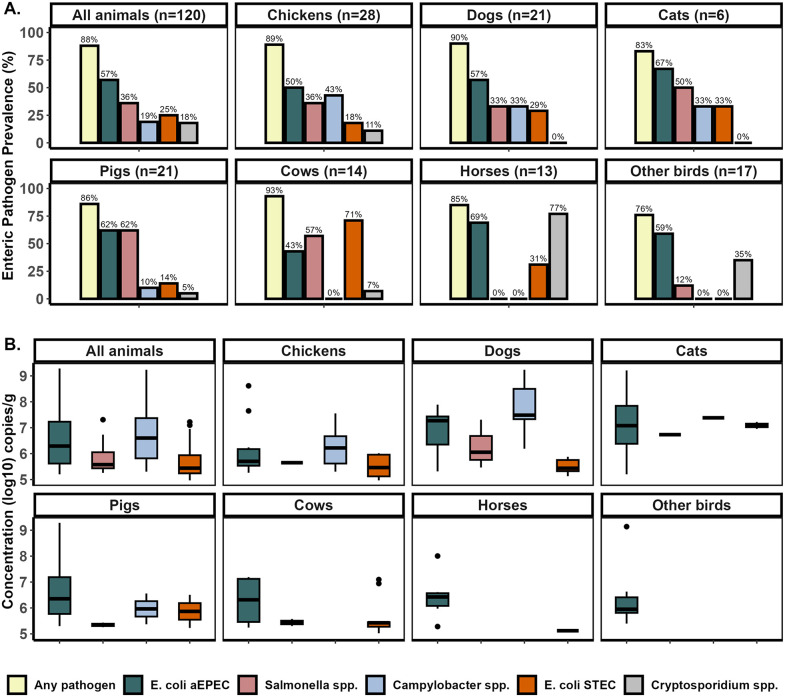
Prevalence and concentration of zoonotic enteric pathogens in animal feces by animal species. **A**. Bar plots show the prevalence (%) of each enteric pathogen detected in animal fecal samples, stratified by site type. Each bar represents the proportion of positive samples for a given pathogen. Pathogens include aEPEC, *Salmonella* sp., *Campylobacter* sp., STEC, and *Cryptosporidium* sp., with “Any pathogen” indicating detection of at least one pathogen in the sample. Bar height and color reflect pathogen-specific prevalence. **B.** Boxplots show the concentration of pathogens (log10 copies/g) detected in animal fecal samples by animal species. Each box represents the interquartile range (IQR) with the median indicated by a horizontal line. *Cryptosporidium* sp. concentrations are not included because the ELISA assay used only allowed for presence/absence detection.

Zoonotic enteric pathogens were frequently detected in animal feces and often were present at high concentrations. Overall, zoonotic pathogens were detected in 106 of 120 samples (88%). The proportion positive for at least one pathogen varied by animal species: chickens 89% (25/28), dogs 90% (19/21), cats 83% (5/6), pigs 86% (18/21), cows 93% (13/14), horses 85% (11/13), and other birds (ducks and parrots) 76% (13/17). The overall prevalence of enteric pathogens varied significantly by animal species (X^2^ (24, N = 120) = 81.12, p < 0.0001). Across all animals, the diarrheagenic *E. coli* pathotype aEPEC was the most common enteric pathogen, detected in 57% of samples and present across all animal species. *Salmonella* sp. was detected in 36% animal feces, primarily in pigs (62%), cows (57%), and cats (50%). The diarrheagenic *E. coli* pathotype STEC was found in 25% of all animal feces, with carriage most frequently detected in cows (71%). *Campylobacter coli/jejuni* was present in 19% of animal feces, predominantly in chickens (43%). *Cryptosporidium* sp. was least common, detected in 18% of animal feces, with horses being notable carriers (77%) (Fig 2A).

Across positive samples, pathogen concentrations spanned 4.97 to 9.29 log_10_ gc/g of feces for all targets. Mean aEPEC concentrations were broadly similar across animal species (chickens 6.16, dogs 6.86, cats 7.14, pigs 6.72, cows 6.27, horses 6.45, and other birds: 6.34 log_10_ gc/g). Cats and dogs showed higher mean concentrations of *Salmonella* sp. (6.73 and 6.27 log_10_ gc/g) and *Campylobacter sp.* (7.75 and 7.38 log_10_ gc/g) than in other animal species (Fig 2B). STEC mean concentrations were also higher in cats (7.09 log_10_ gc/g) than in other animal species. However, pairwise Wilcoxon rank-sum tests indicated no significant differences in pathogen concentrations between animal species for any enteric pathogen (all p-values > 0.05).

**Fig 3:**
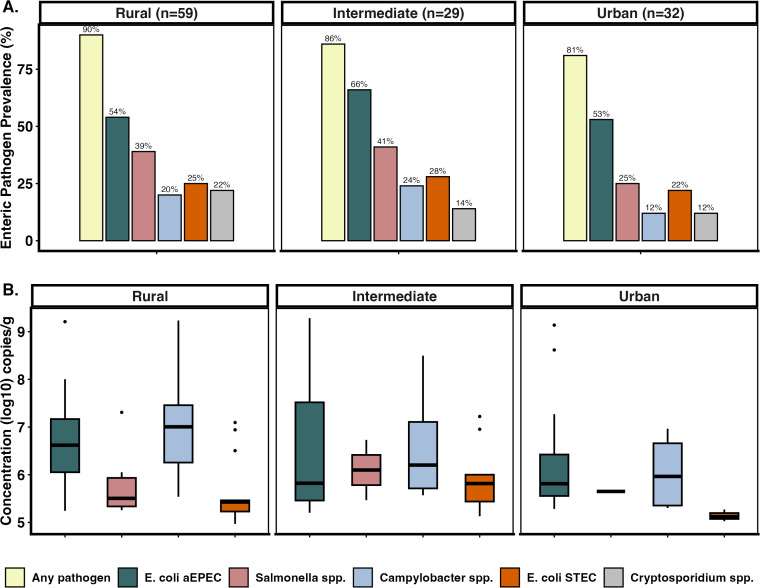
Abundance and coinfection patterns of zoonotic enteric pathogens in animal feces by community. **A.** Bar plots show the prevalence (%) of each enteric pathogen detected in animal fecal samples, stratified by community. Each bar represents the proportion of positive samples for a given pathogen. Pathogens include aEPEC, *Salmonella* sp., *Campylobacter* sp., STEC, and *Cryptosporidium* sp., with “Any pathogen” indicating detection of at least one pathogen in the sample. Bar height and color reflect pathogen-specific prevalence. **B.** Boxplots show the concentration of pathogens (log10 copies/g) detected in animal fecal samples by community. Each box represents the interquartile range (IQR) with the median indicated by a horizontal line. *Cryptosporidium* sp. concentrations are not included because the ELISA assay used only allowed for presence/absence detection.

Detection of potentially zoonotic enteric pathogens was common in animal feces from all communities (Fig 3A), although a non-significant decreasing trend was observed with increasing urbanicity (Chi-square (8, N = 120) = 2.2151, *p* = 0.9737). aEPEC and *Campylobacter sp.* were found at concentrations ~1 log higher in the rural communities compared to the intermediate and urban communities. In contrast, *Salmonella* sp, and *E. coli* STEC had concentrations ~1 log higher in the intermediate community than in rural and urban communities (Fig 3B). We found no significant differences in enteric pathogen concentrations across communities.

**Fig 4:**
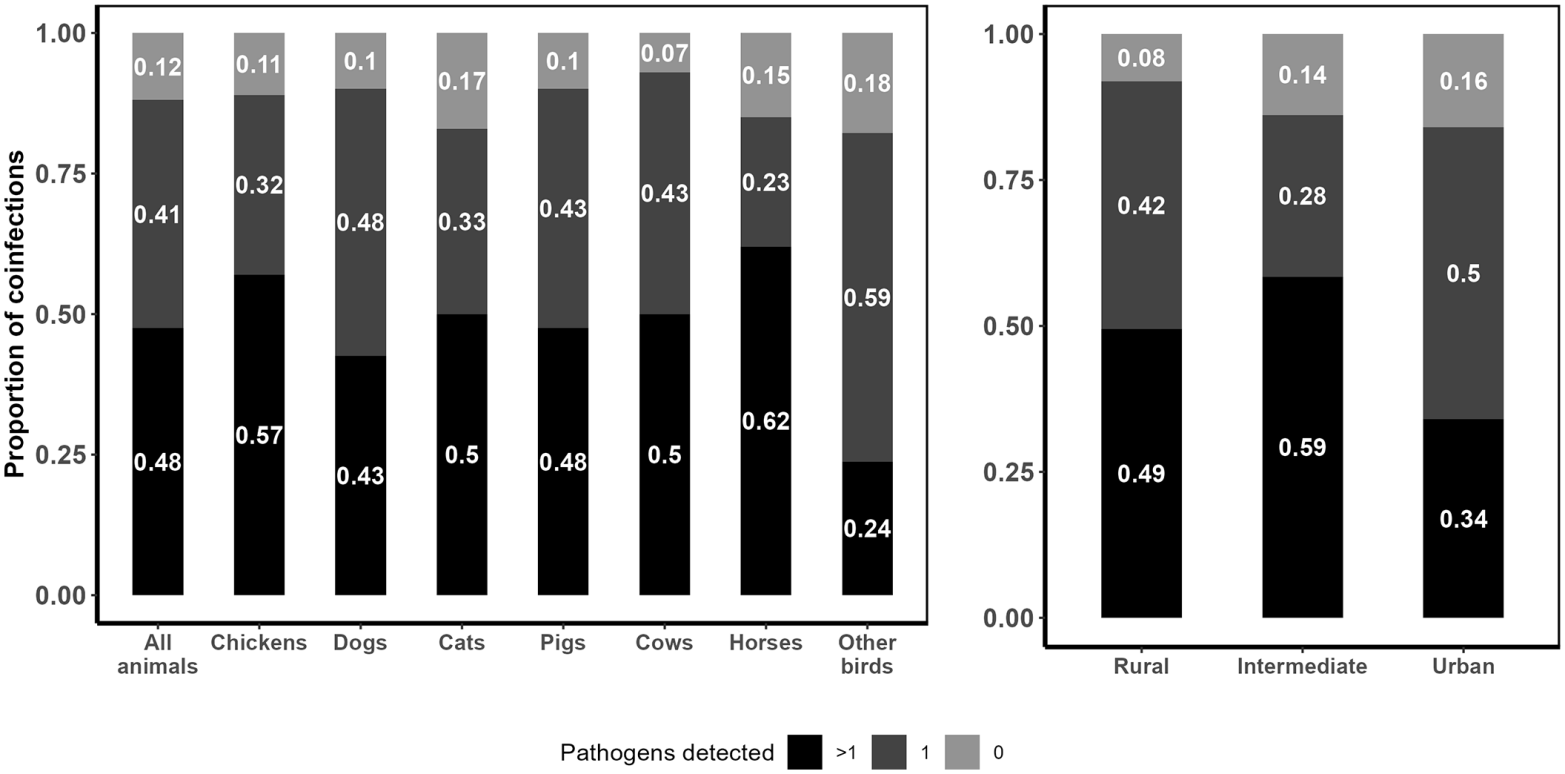
Proportion of coinfections, single infections or no infection with zoonotic enteric pathogens in animal fecal samples by animal species and community. Stacked bar plots depicting the proportion of coinfections (black), single infections (dark gray) and no infections (light gray) in animal fecal samples, stratified by animal species and community.

Coinfections, defined as the detection of more than one enteric pathogen in a single animal fecal sample, occurred in 48% of samples and were common across animal species; chickens (57%) and horses (62%) had the highest coinfection rates (Fig 4A). We identified 11 enteric pathogen co-infection patterns, the most common pattern being *Salmonella* sp. + STEC (21%), followed in order by aEPEC + *Salmonella* sp. (19%), aEPEC + *Cryptosporidium sp.* (16%), and aEPEC + *Campylobacter sp.* (14%) (S4 Table). A probabilistic co-occurrence model indicated that most observed co-occurrences did not deviate from random expectation (S3 Fig). Coinfections were most common in intermediate (59%) and rural (49%) communities, compared to the urban (34%) community. However, these differences were not statistically significant. (Fig 4B).

### (Q2) How are children exposed to animals and their feces?

Widespread animal presence and free-roaming practices created frequent opportunities for child–animal contact. Dogs, cats, and free-range chickens commonly roamed within and between households, often in domestic spaces used for food preparation, laundry, and child play. In one intermediate community, for example, a mother allowed her dog, cat, and chickens to move freely through the backyard, house, and neighborhood. The same areas where animals ate, slept, and defecated were also used by children and caregivers. In contrast, larger animals such as pigs, cows, and horses were generally penned or kept away from houses.

**Fig 5:**
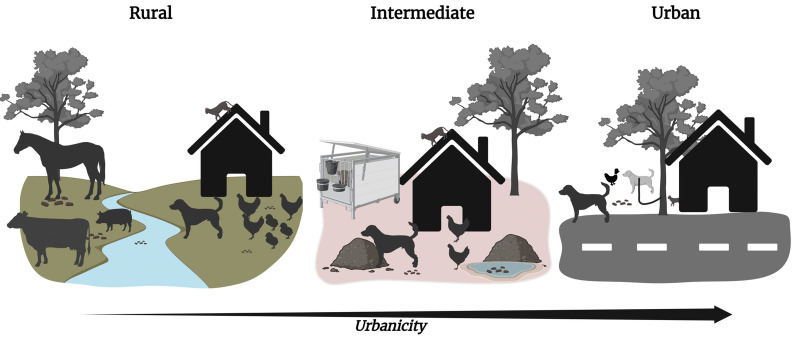
Conceptual illustration of animal fecal contamination across the urban-rural gradient. **Illustration of changes in animal presence and the potential for environmental fecal contamination across the study areas.** In rural areas (left), large animals are kept on farms across the river, while small animals roam freely around households. Animal feces from multiple animal types are commonly observed in the rural household environment. In the intermediate setting (center), both small and large animals are present near homes, though large animals are often kept in containment structures (e.g., animal pens). Animal feces from multiple animal types are commonly observed in the immediate household environment. In urban areas (right), fewer animals are present, and small animals, particularly pets, are more commonly kept in household patios or balconies. Animal feces, predominantly from dogs, are observed in the urban household environment. Created in BioRender. Alban, **V.** (2026) https://BioRender.com/6e5ise9 [30].

Animal presence and roaming patterns varied by community (Fig 5). Urban households generally owned fewer animals and restricted their movement with fenced yards, closed doors, and by tying dogs near entrances as guards. In contrast, intermediate and rural households owned more animals and imposed fewer barriers, allowing freer movement. Urban mothers also cited safety concerns and local norms, such as keeping doors closed, as reasons for limiting animal entry.

Environmental fecal contamination was common outdoors and occasionally indoors, especially from dogs and chickens. Although mothers often downplayed indoor contamination, observations revealed instances in kitchens and living spaces. For example, parrot feces was observed under a chair after apparent cleaning, and dog feces was observed in a kitchen where puppies were kept. Cats were widespread but contributed little visible contamination due to their burying behavior.

Outdoor fecal contamination varied more across communities than indoor contamination, which was generally infrequent. Community practices and household routines appeared to shape these differences. In one rural community, larger animals were kept on farms away from homes, and social norms encouraged families to clean up feces promptly, as neighbors would notice if waste was left in shared spaces. In another rural community, where animals were kept closer to homes and no such norms were described, animal feces were more often observed near households. For example, in one household, a mother fed rice to free-range chickens outside her door each evening to corral them, contributing to feces accumulation near the entrance.

Child–animal and family interactions created frequent exposure opportunities. Children under two often had direct contact with dogs and cats, and sometimes chickens, while playing or crawling in shared indoor and outdoor spaces. Hand-to-mouth and object-to-mouth behaviors were common, and geophagy was occasionally observed. In one rural household, a child was observed chasing chickens and crawling where they had just defecated. The child then put objects, including feces, into their mouth. Such direct contact was less common among urban children, reflecting fewer animals and stricter management practices.

Beyond direct contact, older siblings and caregivers were important indirect exposure pathways, particularly in rural settings. Children frequently had close contact with family members who handled animals or contaminated surfaces. For example, a sibling played with chickens and dogs and then touched the face and mouth of a child under two. Fathers, who often worked with farm animals, also represented potential sources of transmission. In urban households, less frequent animal contact among family members reduced these indirect risks. Across all study communities, children frequently engaged in high-risk behaviors, including crawling and playing on contaminated surfaces, frequent hand-to-mouth contact, and geophagy, providing multiple pathways for potential exposure to zoonotic pathogens.

### (Q3) Which animals may serve as key sources of child exposure to zoonotic pathogens, based on observed behaviors and fecal contamination?

Chickens and dogs emerged as the most likely potential sources of child exposure to zoonotic pathogens, supported by both qualitative observations and microbiological analyses. These animals were major contributors to persistent fecal contamination in household environments, as mothers reported, and observations confirmed, frequent child interactions with them. Free-ranging practices further led to widespread dog and chicken feces around households, heightening exposure risks (Table 2).

**Table 2:** Integrated summary of qualitative observations and microbiological results by community.

	Qualitative methods	Microbiological methods	
Community	Specific observations/behaviors	Enteric pathogen prevalence (%) and coinfections (%)	Enteric pathogen load (mean log10 copies/g of feces)
**Urban**	***Animals*** • Fewer animals and animal types owned (mostly dogs and cats). • Few free-range animals. ***Indoor & outdoor environment*** • Less indoor fecal contamination. • Dog feces outside households are common. ***Children’s behavior*** • Less contact with animals	**Overall:** 81% **Chickens:** 100% **Dogs:** 80% **Coinfections:** 34%	**Overall:** 6.07 **Chickens:** 6.23 **Dogs:** 6.29
**Intermediate**	***Animals*** • Animals frequently roam in indoor and outdoor spaces. • Large animals are kept corralled or contained in a pen near the house. ***Indoor & outdoor environment*** ***•*** Animal feces from multiple animal types observed. ***Children’s behavior*** • Spaces where children play are shared with animals, leading to more interactions.	**Overall:** 86% **Chickens:** 80% **Dogs:** 88% **Coinfections:** 59%	**Overall:** 6.30 **Chickens:** 6.03 **Dogs:** 6.16
**Rural**	***Animals*** • Animals frequently roam in indoor and outdoor spaces. • Large animals are more common but kept on farms, far from homes. ***Indoor & outdoor environment*** ***•*** Animal feces from multiple animal types were observed; however, this varied across rural communities. ***Children’s behavior*** • Spaces where children play are shared with animals, leading to more interactions.	**Overall:** 90% **Chickens:** 92% **Dogs:** 100% **Coinfections:** 49%	**Overall:** 6.38 **Chickens:** 5.93 **Dogs:** 7.23

The qualitative component summarizes observations related to animals, the indoor and outdoor environment, and children’s behaviors. The quantitative component presents the overall prevalence and concentrations (loads) of zoonotic enteric pathogens in animal feces, along with detailed prevalence and concentration data specific to dogs and chickens, two key animal species identified in the study.

Microbiological testing confirmed chickens and dogs as potential exposure sources: 89% of chickens (25/28) and 90% of dogs (19/21) carried at least one enteric pathogen. aEPEC, *Salmonella* sp., and *Campylobacter* sp. were each detected in >30% of samples, with mean concentrations in dogs reaching 6.1 log10 gc/g for *Salmonella* sp. and 7.5 for *Campylobacter* sp. (Fig 2). Co-infections were frequent, affecting 57% of chickens and 43% of dogs, most commonly aEPEC with *Campylobacter* sp. and *Salmonella* sp. with STEC (S4 Table).

## Discussion

This mixed-methods study examines how animal fecal contamination, caregiver and child behaviors, and animal management practices shape young children’s potential exposure to zoonotic enteric pathogens along an urban–rural gradient in northwestern Ecuador. By integrating microbiological data with qualitative observations and maternal reports, we addressed three questions: (Q1) which pathogens are present and at what concentrations, (Q2) how children are exposed to animals and their feces, and (Q3) which animals serve as likely exposure sources. Our findings highlight persistent and widespread pathways that may contribute to environmental exposure, reflecting the interplay of community norms, household practices, and child-environment interactions.

### (Q1) High diversity and high concentrations of zoonotic enteric pathogens highlight a persistent risk

The diversity of zoonotic enteric pathogens detected across animal species, along with high coinfection rates, underscore the potential for simultaneous exposure to multiple pathogens. Some host–pathogen patterns aligned with expectations, such as widespread detection of aEPEC across species [31], and STEC in cattle, consistent with their role as a primary reservoir. [32–35] Other patterns were less expected. For example, in our sample, *Salmonella* sp. was rarely found in chicken feces, despite poultry being a widely recognized reservoir [36,37]. This may reflect the predominance of backyard, low-density, free-ranging poultry systems in our sample, which may reduce transmission compared to intensive operations [38]. However, more concentrated poultry production does occur in the region [39,40], and pathogen dynamics in those settings may differ.

Co-infections involving multiple enteric pathogens were common in animal feces, especially combinations of *Salmonella* sp. with STEC and aEPEC. Such coinfections may exacerbate child health risks by increasing environmental pathogen loads and prolonging shedding [41]. Consistent with this, TaqMan Array Card testing of infant feces at six months showed that 72% (n = 276) of children carried more than two pathogens [42], reinforcing the likelihood of cumulative exposure in early childhood. Similar findings from rural Bangladesh link multiple infections in children to greater stunting risk [43].

Pathogen concentrations in animal feces were consistently high and often exceeded estimated human infectious doses. *Salmonella* sp. reached up to 7 log_10_ gc/g, *Campylobacter* sp. 6.69 log_10_ gc/g, aEPEC 6.53 log_10_ gc/g, and STEC 5.69 log_10_ gc/g, while infectious doses range from 2.8 to 10⁴ cells for *Salmonella* sp. [44], fewer than 10^3^ cells for *Campylobacter* sp. [45], and as few as 50 cells for E. coli STEC [46]. These high concentrations suggest that even brief or indirect contact with animal feces may be sufficient to initiate infection, particularly in young children with developing immune systems and limited hygiene resources. However, risk depends on the degree to which fecal material is attenuated before contact (natural die-off, dilution/dispersion, or handling), which was not quantified.

Although the urban community in our study region generally had better WASH infrastructure and lower livestock densities, these advantages did not markedly reduce the presence of animal feces. Pathogen prevalence and concentrations in animal feces were only modestly lower in the urban community, and the differences were not statistically significant, reinforcing that potential exposure is shaped more by the interplay of community, household, and child-level factors than geography alone. We also found that pathogen distribution varied significantly across animal species, suggesting that the type(s) of animal present or interacted with may be more critical for exposure than location along the urban–rural spectrum.

### (Q2) Daily household, caregiver, and child behaviors sustain multiple exposure pathways

Animal management practices and caregiver and child behaviors created sustained opportunities for zoonotic pathogen exposure. Chickens and dogs were commonly present in both outdoor and indoor spaces, including food preparation, laundry, and child play areas. These are critical household sites where fecal contamination could directly threaten child health, as prior research in Bangladesh [7] and Ethiopia [47] shows associations between animal feces in domestic settings and adverse health outcomes such as environmental enteropathy, stunting, and impaired growth. Observations also revealed that children frequently engaged in high-risk behaviors in spaces where animals were, including crawling and playing on surfaces, frequent hand-to-mouth contact, and geophagy. In northwestern Ecuador [48], children average 2.5 hand-to-mouth contacts and 0.38 oral contacts with soil or garbage per hour, with similar behaviors documented in Bangladesh [49], Zimbabwe [5], and Perú [50]. These patterns emphasize the near-constant nature of environmental contact in early childhood and its role in sustaining high exposure to zoonotic pathogens [2,8,51,52].

Interactions between children and other household members also emerged as an important, yet overlooked, potential transmission pathway [13,53]. Mothers reported keeping infants in animal-free areas, but children had close contact with family members who had handled animals or touched contaminated surfaces, creating opportunities for fecal-oral transmission. Evidence from Ethiopia [54], Bangladesh [55], and northern Ecuador [56] shows *E. coli* and ruminant fecal markers on maternal and child hands, with strong mother–child correlations, supporting the importance of interpersonal pathways in sustaining environmental exposure.

### (Q3) Dogs and chickens are priority animal species for intervention

Among all animal species, dogs and chickens were identified as the most likely potential sources of child exposure to zoonotic pathogens. Chicken feces was ubiquitous and often overlooked in household cleaning routines, likely due to their small size and relatively low odor [10]. These findings mirror patterns in Zambia [57] and Bangladesh [7] where household poultry ownership is common but feces management is not. In contrast, feces from larger animals (e.g., pigs, cows, horses) was less common near households, largely due to containment practices (e.g., penning or tethering). These patterns suggest that free roaming small animals create numerous and repeated opportunities for exposure, supporting the targeting of chickens and dogs in intervention strategies.

A key strength of this study is its convergent mixed-methods design, which integrated in-depth behavioral observation with microbiological testing to provide a more complete picture of everyday exposure pathways. Sampling communities along an urban–rural gradient further supports the relevance of findings for intervention design across diverse settings. Limitations include modest sample sizes and cross-sectional sampling, which limit generalizability and preclude assessment of temporal variation. In addition, our “urban” and “intermediate” strata each included only one community (Esmeraldas city and Borbón, respectively); therefore, differences observed across setting types may reflect community-specific characteristics rather than broader urban or rural patterns. We report population size for each community to clarify their position along the gradient and to aid interpretation and generalizability to similarly sized settings. All data were collected over a relatively narrow window during the dry season. As such, our findings may miss pathogens and exposure pathways that are relevant during the wet season. Seasonality could influence our results in at least two ways: (1) pathogen prevalence and concentrations in animal feces may vary across seasons due to changes in animal diet and husbandry practices, as well as environmental conditions that affect pathogen survival (e.g., temperature and humidity) [58]; and 2) exposure opportunities may shift across seasons. For example, during drier months, animal feces may accumulate and remain accessible in household environments for longer periods, whereas during wetter months, rainfall and runoff could disperse fecal contamination [59]. Notably, we did not assess child infection or fecal shedding, so we cannot directly link pathogen or qualitative data to disease outcomes. In addition, we did not account for attenuation of fecal material before contact (e.g., die-off, dilution, removal) or pathogen viability, so qPCR gene copies may overestimate infectious load. Finally, for *Cryptosporidium* sp., we relied on copro-antigen tests, which are less sensitive than PCR but are practical for screening and epidemiologic studies in the field and in low-resource settings. Thus, conclusions about transmission risk should be interpreted cautiously and as identifying potential exposure pathways rather than confirmed infections.

Children’s potential exposure to zoonotic enteric pathogens in animal feces is shaped by interconnected animal, environmental, microbiological, and behavioral factors. Key risks include the presence of animals in and around households, frequent child–animal contact, and high pathogen burdens in animal feces. Although rural–urban context influences these dynamics, geography alone does not determine risk. Improving child health and reducing stunting may therefore require strategies that go beyond traditional WASH interventions, including restricting animal roaming, promoting regular feces removal with clear community guidance, creating safe animal-free play spaces, and reinforcing caregiver and sibling hygiene practices to limit indirect transmission. Our observational findings also point to more targeted, context-specific solutions. For example, in households where chickens are fed at the front door, shifting feeding away from the main entrances and child play spaces could reduce feces accumulation; in homes where puppies are kept in kitchens or parrots perch in living rooms, designating specific pet areas could lower direct contact; and in rural communities where neighbors already have cleaning systems in place and notice or comment when feces are left in shared spaces, building on these norms with simple tools for feces removal could strengthen routine management of animal feces, including those that are easily overlooked.

## Supporting information

S1 File Methods: Semi-structured interview guide for go-along interviews. (DOCX)

S1 Table Primers and probe sequences for enteric pathogens, gene targets and analytical performance of qPCR assays. (DOCX)

S2 Table MIQE checklist for qPCR assays used to detect zoonotic enteric pathogens in animal feces. (DOCX)

S3 Table Household demographic characteristics by community. (DOCX)

S4 Table Enteric pathogen co-occurrence patterns in different animal types. (DOCX)

S1 Fig Map of the study area. Esmeraldas (urban community), Borbon (intermediate community), and Maldonado, Santo Domingo and Colon (rural communities). Map created in R. **Administrative boundaries were obtained from the GADM database (accessed via the geodata R package; GADM license/terms: gadm.org/license.html). River features were retrieved from OpenStreetMap (via the osmdata R package; OpenStreetMap contributors, Open Database License (ODbL) 1.0: opendatacommons.org/licenses/odbl/). Background country outlines from Natural Earth (public domain: naturalearthdata.com/about/terms-of-use/).
(DOCX)

S2 Fig Mean standard curves of gblock 10-fold serial dilutions generated from individual standard curves per gene. Concentrations ranged from 10 3–10 6 gene copies. error bars represent the standard deviation of Cq values at each concentration.
(DOCX)

S3 Fig Probabilistic co-occurrence model. **Heat map showing the random and non-random enteric pathogen associations determined by the probabilistic co-occurrence model.** Enteric pathogens names are positioned to indicate the column and rows that represent their pairwise relationships with other microorganisms.
(DOCX)

## References

[1] Delahoy MJ, Wodnik B, McAliley L, Penakalapati G, Swarthout J, Freeman MC, et al. Pathogens transmitted in animal feces in low- and middle-income countries. Int J Hyg Environ Health. 2018;221(4):661–76. doi: 10.1016/j.ijheh.2018.03.005 29729998 PMC6013280

[2] Penakalapati G, Swarthout J, Delahoy MJ, McAliley L, Wodnik B, Levy K, et al. Exposure to Animal Feces and Human Health: A Systematic Review and Proposed Research Priorities. Environ Sci Technol. 2017;51(20):11537–52. doi: 10.1021/acs.est.7b02811 28926696 PMC5647569

[3] Cumming O, Curtis V. Implications of WASH Benefits trials for water and sanitation. Lancet Glob Health. 2018;6(6):e613–4. doi: 10.1016/S2214-109X(18)30192-X 29706563

[4] Budge S, Barnett M, Hutchings P, Parker A, Tyrrel S, Hassard F, et al. Risk factors and transmission pathways associated with infant Campylobacter spp. prevalence and malnutrition: A formative study in rural Ethiopia. PLoS ONE. 2020;15: e0232541. doi: 10.1371/journal.pone.0232541 PMC7209302 32384130

[5] George CM, Oldja L, Biswas S, Perin J, Lee GO, Kosek M, et al. Geophagy is associated with environmental enteropathy and stunting in children in rural Bangladesh. Am J Trop Med Hyg. 2015;92(6):1117–24. doi: 10.4269/ajtmh.14-0672 25918214 PMC4458812

[6] Lowenstein C, Vasco K, Sarzosa S, Salinas L, Torres A, Perry MJ, et al. Determinants of Childhood Zoonotic Enteric Infections in a Semirural Community of Quito, Ecuador. Am J Trop Med Hyg. 2020;102(6):1269–78. doi: 10.4269/ajtmh.19-0690 32228797 PMC7253092

[7] George CM, Oldja L, Biswas SK, Perin J, Lee GO, Ahmed S, et al. Fecal Markers of Environmental Enteropathy are Associated with Animal Exposure and Caregiver Hygiene in Bangladesh. Am J Trop Med Hyg. 2015;93(2):269–75. doi: 10.4269/ajtmh.14-0694 26055734 PMC4530746

[8] Pickering AJ, Null C, Winch PJ, Mangwadu G, Arnold BF, Prendergast AJ, et al. The WASH Benefits and SHINE trials: interpretation of WASH intervention effects on linear growth and diarrhoea. Lancet Glob Health. 2019;7(8):e1139–46. doi: 10.1016/S2214-109X(19)30268-2 31303300

[9] Mertens A, Arnold BF, Benjamin-Chung J, Boehm AB, Brown J, Capone D, et al. Effects of water, sanitation, and hygiene interventions on detection of enteropathogens and host-specific faecal markers in the environment: a systematic review and individual participant data meta-analysis. Lancet Planet Health. 2023;7(3):e197–208. doi: 10.1016/S2542-5196(23)00028-1 36889861 PMC10009758

[10] Ercumen A, Pickering AJ, Kwong LH, Arnold BF, Parvez SM, Alam M, et al. Animal Feces Contribute to Domestic Fecal Contamination: Evidence from E. coli Measured in Water, Hands, Food, Flies, and Soil in Bangladesh. Environ Sci Technol. 2017;51(15):8725–34. doi: 10.1021/acs.est.7b01710 28686435 PMC5541329

[11] Mills M, Lee S, Piperata BA, Garabed R, Choi B, Lee J. Household environment and animal fecal contamination are critical modifiers of the gut microbiome and resistome in young children from rural Nicaragua. Microbiome. 2023;11(1):207. doi: 10.1186/s40168-023-01636-5 37715296 PMC10503196

[12] Berendes DM, Yang PJ, Lai A, Hu D, Brown J. Estimation of global recoverable human and animal fecal biomass. Nat Sustain. 2018;1(11):679–85. doi: 10.1038/s41893-018-0167-0 38464867 PMC10922008

[13] Ballard AM, Corozo Angulo B, Laramee N, Pace Gallagher J, Haardörfer R, Freeman MC, et al. Multilevel factors drive child exposure to enteric pathogens in animal feces: A qualitative study in northwestern coastal Ecuador. PLOS Glob Public Health. 2024;4(9):e0003604. doi: 10.1371/journal.pgph.0003604 39292655 PMC11410186

[14] Cohen D, Muhsen K. Vaccines for enteric diseases. Hum Vaccin Immunother. 2019;15(6):1205–14. doi: 10.1080/21645515.2019.1611200 31291174 PMC6663139

[15] Ali HA, Hartner A-M, Echeverria-Londono S, Roth J, Li X, Abbas K, et al. Vaccine equity in low and middle income countries: a systematic review and meta-analysis. Int J Equity Health. 2022;21(1):82. doi: 10.1186/s12939-022-01678-5 35701823 PMC9194352

[16] Wolf J, Hubbard S, Brauer M, Ambelu A, Arnold BF, Bain R, et al. Effectiveness of interventions to improve drinking water, sanitation, and handwashing with soap on risk of diarrhoeal disease in children in low-income and middle-income settings: a systematic review and meta-analysis. Lancet. 2022;400(10345):48–59. doi: 10.1016/S0140-6736(22)00937-0 35780792 PMC9251635

[17] Clasen T, Boisson S, Routray P, Torondel B, Bell M, Cumming O, et al. Effectiveness of a rural sanitation programme on diarrhoea, soil-transmitted helminth infection, and child malnutrition in Odisha, India: a cluster-randomised trial. Lancet Glob Health. 2014;2(11):e645-53. doi: 10.1016/S2214-109X(14)70307-9 25442689

[18] Cumming O, Arnold BF, Ban R, Clasen T, Esteves Mills J, Freeman MC, et al. The implications of three major new trials for the effect of water, sanitation and hygiene on childhood diarrhea and stunting: a consensus statement. BMC Med. 2019;17(1):173. doi: 10.1186/s12916-019-1410-x 31462230 PMC6712663

[19] Lee GO, Eisenberg JNS, Uruchima J, Vasco G, Smith SM, Van Engen A, et al. Gut microbiome, enteric infections and child growth across a rural-urban gradient: protocol for the ECoMiD prospective cohort study. BMJ Open. 2021;11(10):e046241. doi: 10.1136/bmjopen-2020-046241 34686548 PMC8543627

[20] Alban V. Created in Biorender. https://BioRender.com/7j6blpq

[21] Guerrant RL, DeBoer MD, Moore SR, Scharf RJ, Lima AAM. The impoverished gut--a triple burden of diarrhoea, stunting and chronic disease. Nat Rev Gastroenterol Hepatol. 2013;10(4):220–9. doi: 10.1038/nrgastro.2012.239 23229327 PMC3617052

[22] Hennink M, Kaiser BN. Sample sizes for saturation in qualitative research: A systematic review of empirical tests. Soc Sci Med. 2022;292:114523. doi: 10.1016/j.socscimed.2021.114523 34785096

[23] Heymans R, Vila A, van Heerwaarden CAM, Jansen CCC, Castelijn GAA, van der Voort M, et al. Rapid detection and differentiation of Salmonella species, Salmonella Typhimurium and Salmonella Enteritidis by multiplex quantitative PCR. PLoS One. 2018;13(10):e0206316. doi: 10.1371/journal.pone.0206316 30359449 PMC6201931

[24] Liu J, Gratz J, Amour C, Kibiki G, Becker S, Janaki L, et al. A laboratory-developed TaqMan Array Card for simultaneous detection of 19 enteropathogens. J Clin Microbiol. 2013;51(2):472–80. doi: 10.1128/JCM.02658-12 23175269 PMC3553916

[25] Deer DM, Lampel KA, González-Escalona N. A versatile internal control for use as DNA in real-time PCR and as RNA in real-time reverse transcription PCR assays. Lett Appl Microbiol. 2010;50(4):366–72. doi: 10.1111/j.1472-765X.2010.02804.x 20149084

[26] Helmy YA, Krücken J, Nöckler K, von Samson-Himmelstjerna G, Zessin K-H. Comparison between two commercially available serological tests and polymerase chain reaction in the diagnosis of Cryptosporidium in animals and diarrhoeic children. Parasitol Res. 2014;113(1):211–6. doi: 10.1007/s00436-013-3645-3 24221885

[27] McMahon SA, Winch PJ. Systematic debriefing after qualitative encounters: an essential analysis step in applied qualitative research. BMJ Glob Health. 2018;3(5):e000837. doi: 10.1136/bmjgh-2018-000837 30233833 PMC6135453

[28] Bustin SA, Benes V, Garson JA, Hellemans J, Huggett J, Kubista M, et al. The MIQE guidelines: minimum information for publication of quantitative real-time PCR experiments. Clin Chem. 2009;55(4):611–22. doi: 10.1373/clinchem.2008.112797 19246619

[29] Griffith DM, Veech JA, Marsh CJ. cooccur: Probabilistic Species Co-Occurrence Analysis inR. J Stat Soft. 2016;69(Code Snippet 2). doi: 10.18637/jss.v069.c02

[30] Alban V. Created in Biorender. https://BioRender.com/6e5ise9

[31] Vasco K, Graham JP, Trueba G. Detection of Zoonotic Enteropathogens in Children and Domestic Animals in a Semirural Community in Ecuador. Appl Environ Microbiol. 2016;82(14):4218–24. doi: 10.1128/aem.00795-16 27208122 PMC4959199

[32] Trueba G, Garcés V, V VB, Colman RE, Seymour M, Vogler AJ, et al. Escherichia coli O157:H7 in Ecuador: animal reservoirs, yet no human disease. Vector Borne Zoonotic Dis. 2013;13(5):295–8. doi: 10.1089/vbz.2012.1128 23473224

[33] Cerqueira AM, Guth BE, Joaquim RM, Andrade JR. High occurrence of Shiga toxin-producing Escherichia coli (STEC) in healthy cattle in Rio de Janeiro State, Brazil. Vet Microbiol. 1999;70(1–2):111–21. doi: 10.1016/s0378-1135(99)00123-6 10591502

[34] Padola NL, Sanz ME, Blanco JE, Blanco M, Blanco J, Etcheverria AI, et al. Serotypes and virulence genes of bovine Shigatoxigenic Escherichia coli (STEC) isolated from a feedlot in Argentina. Vet Microbiol. 2004;100(1–2):3–9. doi: 10.1016/S0378-1135(03)00127-5 15135507

[35] Hussein HS, Sakuma T. Prevalence of shiga toxin-producing Escherichia coli in dairy cattle and their products. J Dairy Sci. 2005;88(2):450–65. doi: 10.3168/jds.s0022-0302(05)72706-5 15653509

[36] Thames HT, Sukumaran AT. A Review of Salmonella and Campylobacter in Broiler Meat: Emerging Challenges and Food Safety Measures. Foods. 2020;9(6):776. doi: 10.3390/foods9060776 32545362 PMC7353592

[37] Tariq S, Samad A, Hamza M, Ahmer A, Muazzam A, Ahmad S, et al. Salmonella in Poultry; An Overview. ijmdsa. 2022;1(1):80–4. doi: 10.47709/ijmdsa.v1i1.1706

[38] Oh H, Yoon Y, Yoon J-W, Oh S-W, Lee S, Lee H. Salmonella Risk Assessment in Poultry Meat from Farm to Consumer in Korea. Foods. 2023;12(3):649. doi: 10.3390/foods12030649 36766177 PMC9914641

[39] Hedman HD, Eisenberg JNS, Vasco KA, Blair CN, Trueba G, Berrocal VJ, et al. High Prevalence of Extended-Spectrum Beta-Lactamase CTX-M-Producing Escherichia coli in Small-Scale Poultry Farming in Rural Ecuador. Am J Trop Med Hyg. 2019;100(2):374–6. doi: 10.4269/ajtmh.18-0173 30457098 PMC6367627

[40] Guo X, Stedtfeld RD, Hedman H, Eisenberg JNS, Trueba G, Yin D, et al. Antibiotic Resistome Associated with Small-Scale Poultry Production in Rural Ecuador. Environ Sci Technol. 2018;52(15):8165–72. doi: 10.1021/acs.est.8b01667 29944836

[41] Ruby T, McLaughlin L, Gopinath S, Monack D. Salmonella’s long-term relationship with its host. FEMS Microbiol Rev. 2012;36(3):600–15. doi: 10.1111/j.1574-6976.2012.00332.x 22335190

[42] Jesser KJ, Zhou NA, Hemlock C, Miller-Petrie MK, Contreras JD, Ballard A, et al. Environmental Exposures Associated with Enteropathogen Infection in Six-Month-Old Children Enrolled in the ECoMiD Cohort along a Rural-Urban Gradient in Northern Ecuador†. Environ Sci Technol. 2025;59(1):103–18. doi: 10.1021/acs.est.4c07753 39807583 PMC11740902

[43] George CM, Burrowes V, Perin J, Oldja L, Biswas S, Sack D, et al. Enteric Infections in Young Children are Associated with Environmental Enteropathy and Impaired Growth. Trop Med Int Health. 2018;23(1):26–33. doi: 10.1111/tmi.13002 29121442

[44] Doyle MP, Beuchat LR. Food microbiology: fundamentals and frontiers. 3rd ed. Washington, D.C.: ASM Press. 2007.

[45] Tribble DR, Baqar S, Scott DA, Oplinger ML, Trespalacios F, Rollins D, et al. Assessment of the duration of protection in Campylobacter jejuni experimental infection in humans. Infect Immun. 2010;78(4):1750–9. doi: 10.1128/IAI.01021-09 20086085 PMC2849408

[46] Karmali MA. Infection by Shiga toxin-producing Escherichia coli: an overview. Mol Biotechnol. 2004;26(2):117–22. doi: 10.1385/MB:26:2:117 14764937

[47] Headey D, Hirvonen K. Is Exposure to Poultry Harmful to Child Nutrition? An Observational Analysis for Rural Ethiopia. PLoS One. 2016;11(8):e0160590. doi: 10.1371/journal.pone.0160590 27529178 PMC4986937

[48] Sosa-Moreno A, Lee GO, Van Engen A, Sun K, Uruchima J, Kwong LH, et al. Characterizing Behaviors Associated with Enteric Pathogen Exposure among Infants in Rural Ecuador through Structured Observations. Am J Trop Med Hyg. 2022;106: 1747–56. doi: 10.4269/ajtmh.21-1099 35405653 PMC9209906

[49] Ngure FM, Humphrey JH, Mbuya MNN, Majo F, Mutasa K, Govha M, et al. Formative research on hygiene behaviors and geophagy among infants and young children and implications of exposure to fecal bacteria. Am J Trop Med Hyg. 2013;89(4):709–16. doi: 10.4269/ajtmh.12-0568 24002485 PMC3795101

[50] Marquis GS, Ventura G, Gilman RH, Porras E, Miranda E, Carbajal L, et al. Fecal contamination of shanty town toddlers in households with non-corralled poultry, Lima, Peru. Am J Public Health. 1990;80(2):146–9. doi: 10.2105/ajph.80.2.146 2297055 PMC1404601

[51] Ballard AM, Laramee N, Haardörfer R, Freeman MC, Levy K, Caruso BA. Measurement in the study of human exposure to animal feces: A systematic review and audit. Int J Hyg Environ Health. 2023;249:114146. doi: 10.1016/j.ijheh.2023.114146 36868140 PMC10044406

[52] Zambrano LD, Levy K, Menezes NP, Freeman MC. Human diarrhea infections associated with domestic animal husbandry: a systematic review and meta-analysis. Trans R Soc Trop Med Hyg. 2014;108(6):313–25. doi: 10.1093/trstmh/tru056 24812065 PMC4023907

[53] Ballard AM, Haardörfer R, Angulo BC, Freeman MC, Eisenberg JNS, Lee GO, et al. The development and validation of a survey to measure fecal-oral child exposure to zoonotic enteropathogens: The FECEZ Enteropathogens Index. PLOS Glob Public Health. 2024;4(9):e0002690. doi: 10.1371/journal.pgph.0002690 39255298 PMC11386431

[54] Deblais L, Ahmedo BU, Ojeda A, Mummed B, Wang Y, Mekonnen YT, et al. Assessing fecal contamination from human and environmental sources using Escherichia coli as an indicator in rural eastern Ethiopian households-a cross-sectional study from the EXCAM project. Front Public Health. 2025;12:1484808. doi: 10.3389/fpubh.2024.1484808 39835307 PMC11743629

[55] Fuhrmeister ER, Ercumen A, Pickering AJ, Jeanis KM, Ahmed M, Brown S, et al. Predictors of Enteric Pathogens in the Domestic Environment from Human and Animal Sources in Rural Bangladesh. Environ Sci Technol. 2019;53(17):10023–33. doi: 10.1021/acs.est.8b07192 31356066 PMC6727619

[56] Jesser KJ, Alban V, Lobos AE, Gallard-Góngora J, Trueba G, Lee GO, et al. Microbial source tracking of human and animal fecal contamination in Ecuadorian households. bioRxiv. 2025;:2025.08.22.671888. doi: 10.1101/2025.08.22.671888 41405239 PMC12838389

[57] Reid B, Seu R, Orgle J, Roy K, Pongolani C, Chileshe M, et al. A Community-Designed Play-Yard Intervention to Prevent Microbial Ingestion: A Baby Water, Sanitation, and Hygiene Pilot Study in Rural Zambia. Am J Trop Med Hyg. 2018;99(2):513–25. doi: 10.4269/ajtmh.17-0780 29869596 PMC6090365

[58] Chao DL, Roose A, Roh M, Kotloff KL, Proctor JL. The seasonality of diarrheal pathogens: A retrospective study of seven sites over three years. PLoS Negl Trop Dis. 2019;13(8):e0007211. doi: 10.1371/journal.pntd.0007211 31415558 PMC6711541

[59] Bhavnani D, Goldstick JE, Cevallos W, Trueba G, Eisenberg JNS. Impact of rainfall on diarrheal disease risk associated with unimproved water and sanitation. Am J Trop Med Hyg. 2014;90(4):705–11. doi: 10.4269/ajtmh.13-0371 24567318 PMC3973516

