# Supplementary material for: Child exposure to animal feces and zoonotic pathogens in northwest Ecuador: A mixed-methods study
Source: PLoS Negl Trop Dis. 2026 Feb 23;20(2):e0014019. doi: 10.1371/journal.pntd.0014019 (PMC12956073; doi:10.1371/journal.pntd.0014019)
Supplement: S1 File — (DOCX) [file pntd.0014019.s001.docx]

**SI Methods:** Semi-structured interview guide for go-along interviews

Interviewer Introduction: We would like to learn about animals and the environment in your community. The interview will focus on your daily activities and work in and around your home, on your property/farm, or in other places. We will ask about animals and how you care for them and other related topics. We will ask you to show us the activities and tasks you do during a normal day. We will ask questions so that you can tell us what you are thinking while you look around and walk through your surroundings. These questions are more like a conversation. There are no right or wrong answers; we are simply looking for information about your opinions and experiences. This activity will last 45 minutes or longer.

*Instructions for interviewer: If possible, go to the places mentioned at the moment the person is talking about them.*

Interviewer: We will start with questions about a typical day for you and your child.

1. What do you and your child do on a typical day?
   1. Where do you go?
   2. Where does the child spend most of the day?
   3. Where are you?

**Animal ownership**

1. What animals do you have?
   1. Have you always had these animals?
   2. Are there times you have other types of animals?
   3. Why do you have these animals? For consumption? For income? For trade/barter? Other reasons?
   4. What animals are typical in your community?
   5. What animals do your neighbors and friends have?
2. Where are the animals’ habitats?
   1. Where do they spend the day? In the house? Outside? What is normal for you?
   2. Where do the animals sleep? In the house? Outside?
3. How are the animals cared for daily?
4. Who cleans the animals’ habitats?
   1. How are the habitats cleaned?
   2. What type of products are used?
5. Who cleans the animal feces?
   1. What happens after cleaning?
   2. What do you do with the animal feces?
6. How do you care for the animals daily?
7. During a typical day, what animals are inside your house? What about outside?
   1. Are they your animals?
   2. Are there other animals that belong to other community members’ animals?
   3. Are there natural or wild animals?
   4. What happens when an animal enters your house? What is typical?
   5. Does your family eat with animals inside the house?

*Ask the participant to walk with you to locations. They can perform the tasks or activities if they wish. They can explain how they feed the animals, clean them, where the animals go, etc.*

*The interviewer should ask probing questions about the animals, other potential exposures, and daily activities, as needed.*

**Animal interactions**

1. Does your child interact with your animals? Do they interact with animals that are not yours?
   1. How do they interact with animals?
   2. What do you think about this?
2. Are there animals that are good for children to have contact with?
   1. Are there animals that are not good for children to have contact with?
   2. Why?

**Final thoughts**

1. Is there anything else you would like to say? Thank you for your time!
