# Supplementary material for: Child exposure to animal feces and zoonotic pathogens in northwest Ecuador: A mixed-methods study
Source: PLoS Negl Trop Dis. 2026 Feb 23;20(2):e0014019. doi: 10.1371/journal.pntd.0014019 (PMC12956073; doi:10.1371/journal.pntd.0014019)
Supplement: S1 Table — (DOCX) [file pntd.0014019.s002.docx]

## **S1 Table.** Primers and probe sequences for enteric pathogens, gene targets and analytical performance of qPCR assays.

| Enteric pathogen | | Gene | | Primers | Efficiency (%)^1^ | R2^1^ |  | Slope | Y-intercept | LOD^2^  *(equiv. no. of copies)* | Reproducibility^3^  *(Low- and high-concn. CV (%))* |
| --- | --- | --- | --- | --- | --- | --- | --- | --- | --- | --- | --- |
| *Salmonella* sp. | | *invA*^12^ | **F:** GCTGCTTTCTCTACTTAAC  **R:** GTAATGGAATGACGAACAT  **P:** FAM-CATCACCATTAGTACCAGAATCAGT-BHQ1 | | 94.57 | 0.99 |  | -3.46 | 46.42 | 10^5^ (100) | 0.99-1.42 |
| *Campylobacter* sp. | | *cadF*^13^ | **F:** CTGCTAAACCATAGAAATAAAATTTCTCAC  **R:** CTTTGAAGGTAATTTAGATATGGATAATCG  **P:** FAM-CATTTTGACGATTTTTGGCTTGA-BHQ1 | | 93.46 | 0.99 |  | -3.49 | 48.02 | 10^5^ (100) | 0.54-1.05 |
| *E. coli* (aEPEC) | | *eae*^13^ | **F:** CATTGATCAGGATTTTTCTGGTGATA  **R:** CTCATGCGGAAATAGCCGTTA  **P:** FAM-ATACTGGCGAGACTATTTCAA-BHQ1 | | 95.02 | 0.99 |  | -3.45 | 47.26 | 10^5^ (100) | 0.72-2.90 |
| *E. coli* (aEPEC) | | *bfpA*^13^ | **F:** TGGTGCTTGCGCTTGCT  **R:** CGTTGCGCTCATTACTTCTG  **P:** FAM-CAGTCTGCGTCTGATTCCAA-BHQ1 | | 96.72 | 0.99 |  | -3.40 | 44.71 | 10^5^ (100) | 0.39-2.93 |
| *E. coli*  (STEC) | | *sxt1*^13^ | **F:** ACTTCTCGACTGCAAAGACGTATG  **R:** ACAAATTATCCCCTGWGCCACTATC  **P:** FAM-CTCTGCAATAGGTACTCCA-BHQ1 | | 93.3 | 1.00 |  | -3.50 | 48.00 | 10^5^ (100) | 0.49-1,00 |
| *E. coli*  (STEC) | | *stx2*^13^ | **F:** CCACATCGGTGTCTGTTATTAACC  **R:** GGTCAAAACGCGCCTGATAG  **P:** FAM-TTGCTGTGGATATACGAGG-BHQ1 | | 92.24 | 1.00 |  | -3.52 | 46.21 | 10^5^ (100) | 0.22-1.26 |
|  | | IC^14^ | **F:** CTAACCTTCGTGATGAGCAATCG  **R:** GATCAGCTACGTGAGGTCCTAC | |  |  |  |  |  |  |  |
|  | ***All analysis was based on four standard curves per target  ^1^ The linearity range was 10^3^ to 10^6^ copy numbers per reaction for all targets  ^2^ LOD, copy number of the artificial template per gram of feces, equiv. no. of copies (equivalent copy numbers per 1 µL of volume).  ^3^ Coefficients of variance (CVs) at both low and high concentrations are shown. | | | | | | | | | | |
