# Supplementary material for: Child exposure to animal feces and zoonotic pathogens in northwest Ecuador: A mixed-methods study
Source: PLoS Negl Trop Dis. 2026 Feb 23;20(2):e0014019. doi: 10.1371/journal.pntd.0014019 (PMC12956073; doi:10.1371/journal.pntd.0014019)
Supplement: S2 Table — (DOCX) [file pntd.0014019.s003.docx]

## **S2 Table:** MIQE checklist for qPCR assays used to detect zoonotic enteric pathogens in animal feces

| **MIQE Item** | **How it was addressed in this study** |
| --- | --- |
| **Sample storage and extraction kit** | Animal fecal samples were collected into sterile containers, aliquoted, flash-frozen in liquid nitrogen within 6 h, transported on dry ice, and stored at −80 °C until extraction. Genomic DNA was extracted using the PowerSoil DNA Isolation Kit according to the manufacturer’s instructions. No additional storage buffer was used. |
| **Primer sequences** | Primer and probe sequences, target genes and references are reported in S1 Table |
| **Primer, probe, Mg²⁺ and dNTP concentrations** | Primer and probe concentrations as above (1 μM primers, 0.1 μM probe). Mg²⁺ and dNTP concentrations are as supplied in the 2× TaqMan® Universal PCR Master Mix. |
| **Polymerase identity and concentration** | Taq DNA polymerase is provided within the TaqMan Universal PCR Master Mix (Applied Biosystems); concentration per reaction follows manufacturer’s formulation. |
| **Complete reaction conditions** | qPCR reactions were run in 20 μL total volume containing 10 μL 2× TaqMan Universal PCR Master Mix, 1 μM of each forward and reverse primer, 0.1 μM probe, and 4 μL of DNA template. |
| **Procedure and instrumentation** | Reactions were run on a CFX96 real-time PCR system. Plates included duplicate 10-fold standard curves (10⁶–10² gene copies per reaction), samples, no-template controls (NTCs), and negative extraction controls. Cycling conditions: 50 °C for 2 min, 95 °C for 10 min, followed by 40 cycles of 95 °C for 15 s and 55 °C or 60 °C for 1 min (depending on assay) |
| **qPCR analysis program and Cq determination** | qPCR data were exported from the instrument software and analyzed using standard Cq threshold settings. Gene abundance was quantified relative to the mean standard curve from four runs and processed following MIQE recommendations. |
| **Number of replicates and repeatability** | All standards and samples were run in duplicate reactions. DQ required amplification in both replicates and a mean Cq within the standard curve range and above LoQ. DQ and DNQ were considered positive for prevalence analyses; ND were considered negative. NTCs and NECs showed no amplification ≤ 40 cycles in all runs, supporting assay repeatability and absence of contamination. |
| **Calibration curves (slope, intercept, efficiency, R², range)** | Calibration curves were generated from 10-fold serial dilutions of a gBlock synthetic standard containing all target sequences (10⁶–10² gene copies per reaction). Mean slope, y-intercept, PCR efficiency, R², and linear dynamic range for each assay are S1 Table and S1 Fig. |
| **Evidence for limit of detection (LoD)** | LoD for each assay was defined as the lowest standard concentration with standard deviation < 1 Cq across replicates and ≥ 95% detection. LoD values are reported in S1 Table. |
| **Limit of quantification (LoQ)** | LoQ was calculated from the LoD Cq value and its standard deviation (σ) as Cq_LoQ = Cq_LoD − 2σ_LoD. Samples with mean Cq between the highest and lowest standards and below the LoQ threshold were considered detectable and quantifiable (DQ). |
| **Normalization method** | Absolute quantification was performed using standard curves; no housekeeping gene normalization was applied because the outcome is gene copies per reaction. |
