# Supplementary material for: Child exposure to animal feces and zoonotic pathogens in northwest Ecuador: A mixed-methods study
Source: PLoS Negl Trop Dis. 2026 Feb 23;20(2):e0014019. doi: 10.1371/journal.pntd.0014019 (PMC12956073; doi:10.1371/journal.pntd.0014019)
Supplement: S3 Table — (DOCX) [file pntd.0014019.s004.docx]

## **S3 Table:** Household demographic characteristics by community.

|  | Total  (n=35) | Rural  (n=10) | Intermediate  (n=15) | Urban  (n=10) |
| --- | --- | --- | --- | --- |
| Mean maternal age in years (SD) | 26 (6) | 24 (6) | 27 (6) | 26 (5) |
| Mean child age in months (SD) | 13 (6) | 14 (5) | 12 (6) | 13 (7) |
| Child sex (%) |  |  |  |  |
| *Male* | 13 (37.1) | 3 (30.0) | 6 (40.0) | 4 (40.0) |
| *Female* | 22 (62.9) | 7 (70.0) | 9 (60.0) | 6 (60.0) |
| Drinking water source (%) |  |  |  |  |
| *Purchased water* | 16 (45.7) | 0 (0.0) | 11 (73.3) | 5 (50.0) |
| *Well or tubewell water* | 1 (2.9) | 0 (0.0) | 1 (6.7) | 0 (0.0) |
| *Piped water* | 7 (20.0) | 0 (0.0) | 2 (13.3) | 5 (50.0) |
| *Rainwater* | 8 (22.9) | 7 (70.0) | 1 (6.7) | 0 (0.0) |
| *River water* | 3 (8.6) | 3 (30.0) | 0 (0.0) | 0 (0.0) |
| Bathroom access (%) |  |  |  |  |
| *Household toilet or latrine* | 29 (82.9) | 5 (50.0) | 14 (93.3) | 10 (100.0) |
| *Public or community latrine* | 2 (5.7) | 2 (20.0) | 0 (0.0) | 0 (0.0) |
| *Neighbor’s toilet or latrine* | 3 (8.6) | 3 (30.0) | 0 (0.0) | 0 (0.0) |
| *Hole or pit* | 1 (2.9) | 0 (0.0) | 1 (6.7) | 0 (0.0) |
