# Supplementary material for: Child exposure to animal feces and zoonotic pathogens in northwest Ecuador: A mixed-methods study
Source: PLoS Negl Trop Dis. 2026 Feb 23;20(2):e0014019. doi: 10.1371/journal.pntd.0014019 (PMC12956073; doi:10.1371/journal.pntd.0014019)
Supplement: S4 Table — (DOCX) [file pntd.0014019.s005.docx]

**S4 Table:** Enteric pathogen co-occurrence patterns in different animal types.

| Co-ocurrence patterns | Animal type | | | | | | | | |
| --- | --- | --- | --- | --- | --- | --- | --- | --- | --- |
|  | **Cats** | **Chickens** | **Cows** | **Dogs** | **Ducks** | **Horses** | **Parrots** | **Pigs** | **Total**  **n**  **(%)** |
| aEPEC *+*  *Cryptosporidium* sp. | 0 | 1 | 0 | 0 | 2 | 6 | 0 | 0 | 9 (16%) |
| aEPEC *+*  *Salmonella spp.* | 0 | 2 | 1 | 1 | 1 | 0 | 0 | 6 | 11 (19%) |
| aEPEC *+*  *Campylobacter* sp. | 0 | 4 | 0 | 3 | 0 | 0 | 0 | 1 | 8 (14%) |
| *Salmonella spp. +*  *Campylobacter* sp. | 1 | 2 | 0 | 1 | 0 | 0 | 0 | 0 | 4 (7%) |
| *Salmonella spp. +*  *Cryptosporidium* sp. | 0 | 1 | 0 | 0 | 0 | 0 | 0 | 0 | 1 (2%) |
| *Salmonella spp. +*  STEC | 1 | 2 | 5 | 2 | 0 | 0 | 0 | 2 | 12 (21%) |
| *Campylobacter* sp.*+*  STEC | 0 | 1 | 0 | 0 | 0 | 0 | 0 | 0 | 1 (2%) |
| *Cryptosporidium* sp.*+*  STEC | 0 | 1 | 1 | 0 | 0 | 2 | 0 | 0 | 4 (7%) |
| *Salmonella spp. +* aEPEC *+ Cryptosporidium* sp. | 0 | 0 | 0 | 0 | 1 | 0 | 0 | 1 | 2 (4%) |
| *Salmonella spp. + Campylobacter* sp. *+* aEPEC | 0 | 1 | 0 | 1 | 0 | 0 | 0 | 0 | 2 (4%) |
| *Salmonella spp. + Campylobacter* sp. *+* STEC | 1 | 1 | 0 | 1 | 0 | 0 | 0 | 0 | 3 (5%) |
| Total  n (%) | 3 (5%) | 16  (28%) | 7 (12%) | 9 (16%) | 4  (7%) | 8  (14%) | 0  (0%) | 10 (16%) | 57 |
