# Supplementary material for: Child exposure to animal feces and zoonotic pathogens in northwest Ecuador: A mixed-methods study
Source: PLoS Negl Trop Dis. 2026 Feb 23;20(2):e0014019. doi: 10.1371/journal.pntd.0014019 (PMC12956073; doi:10.1371/journal.pntd.0014019)
Supplement: S1 Fig — Esmeraldas (urban community), Borbon (intermediate community), and Maldonado, Santo Domingo and Colon (rural communities). Map created in R. **Administrative boundaries were obtained from the GADM database (accessed via the geodata R package; GADM license/terms: gadm.org/license.html). River features were retrieved from OpenStreetMap (via the osmdata R package; OpenStreetMap contributors, Open Database License (ODbL) 1.0: opendatacommons.org/licenses/odbl/). Background country outlines from Natural Earth (public domain: naturalearthdata.com/about/terms-of-use/). (DOCX) [file pntd.0014019.s006.docx]

**S1 Fig.** Map of the study area. Esmeraldas (urban community), Borbon (intermediate community), and Maldonado, Santo Domingo and Colon (rural communities).

**
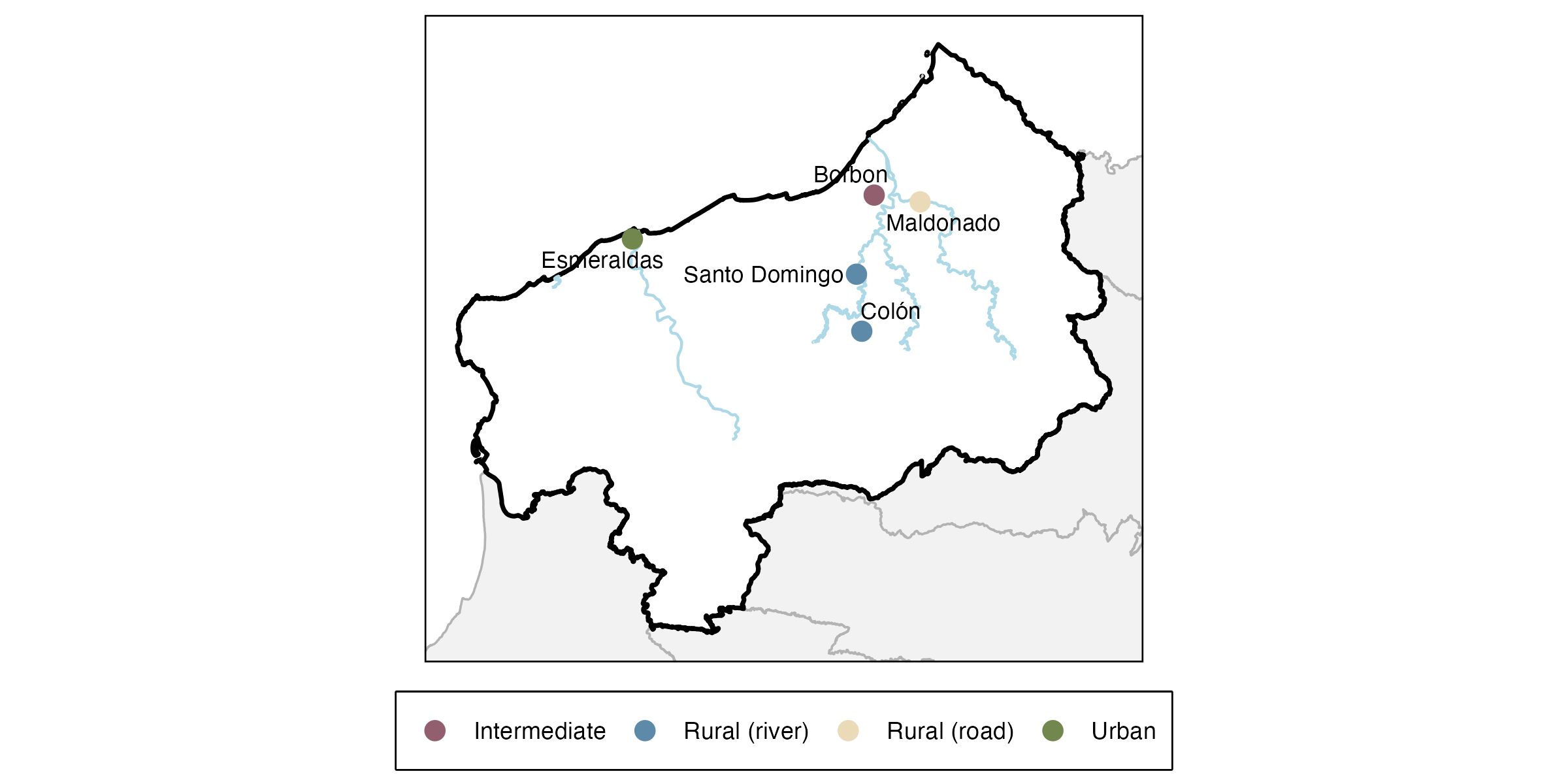
**
