# Supplementary material for: Child exposure to animal feces and zoonotic pathogens in northwest Ecuador: A mixed-methods study
Source: PLoS Negl Trop Dis. 2026 Feb 23;20(2):e0014019. doi: 10.1371/journal.pntd.0014019 (PMC12956073; doi:10.1371/journal.pntd.0014019)

**S2 Fig:** Mean standard curves of gblock 10-fold serial dilutions generated from individual standard curves per gene**.** Concentrations ranged from 10 3 to 10 6 gene copies. error bars represent the standard deviation of Cq values at each concentration.


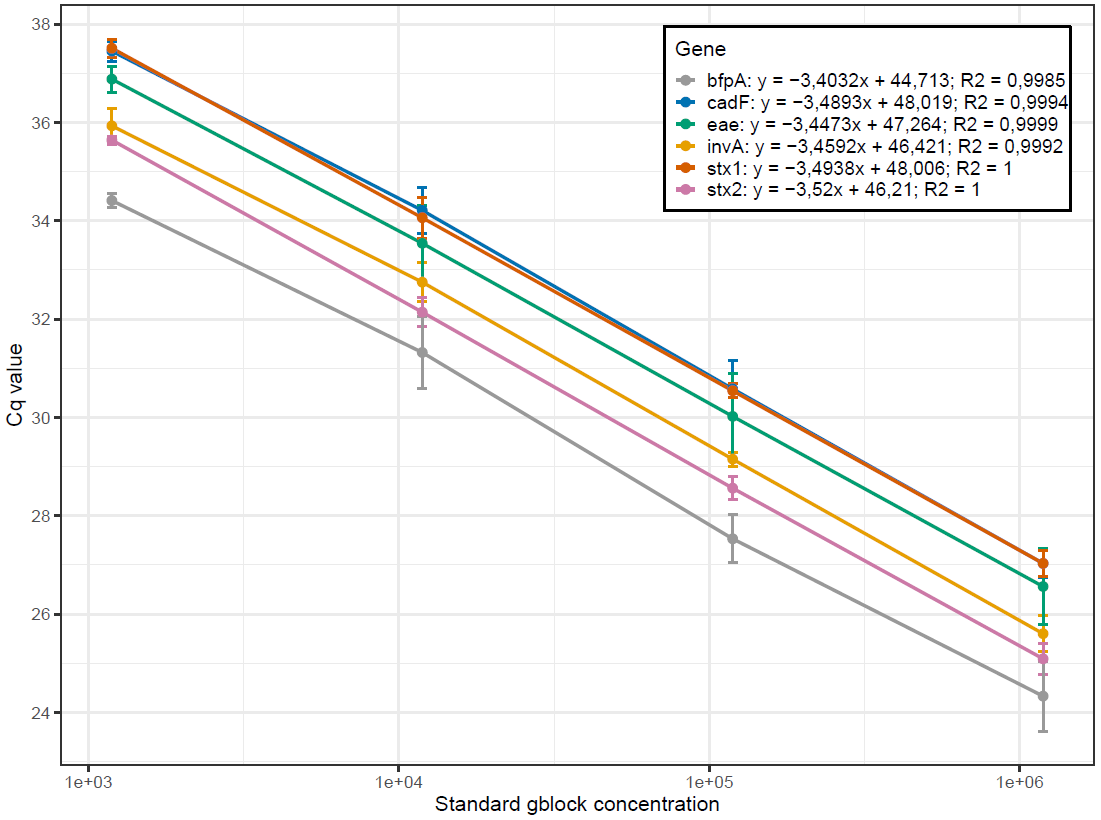

Supplement: S2 Fig — Concentrations ranged from 10 3–10 6 gene copies. error bars represent the standard deviation of Cq values at each concentration. (DOCX) [file pntd.0014019.s007.docx]
