# Supplementary material for: Child exposure to animal feces and zoonotic pathogens in northwest Ecuador: A mixed-methods study
Source: PLoS Negl Trop Dis. 2026 Feb 23;20(2):e0014019. doi: 10.1371/journal.pntd.0014019 (PMC12956073; doi:10.1371/journal.pntd.0014019)

**S3 Fig.** Probabilistic co-occurrence model. Heat map showing the random and non-random enteric pathogen associations determined by the probabilistic co-occurrence model. Enteric pathogens names are positioned to indicate the column and rows that represent their pairwise relationships with other microorganisms.


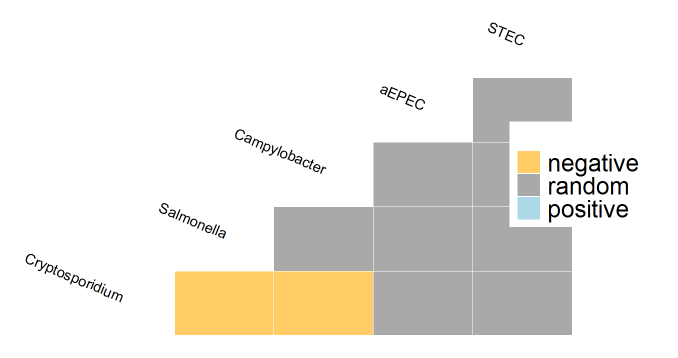

Supplement: S3 Fig — Heat map showing the random and non-random enteric pathogen associations determined by the probabilistic co-occurrence model. Enteric pathogens names are positioned to indicate the column and rows that represent their pairwise relationships with other microorganisms. (DOCX) [file pntd.0014019.s008.docx]
